# Supplementary material for: Biomonitoring of Indoor Air Fungal or Chemical Toxins with Caenorhabditis elegans nematodes
Source: Pathogens. 2023 Jan 19;12(2):161. doi: 10.3390/pathogens12020161 (PMC9964051; doi:10.3390/pathogens12020161)
Supplement: Supplementary file 1 [file pathogens-12-00161-s001.zip › pathogens-2138986-supplementary.pdf]

Paavanen-Huhtala et al.

Biomonitoring of indoor air fungal or chemical toxins with *Caenorhabditis elegans* nematodes

Supplementary data

Supplementary Table 1. Known biological activities of the mycotoxins produced by the fungi listed in Table 2.

**Toxin**	**Biological activity**	**Fungi producing them**
Averufin	Inhibits mitochondrial respiration [63]	*Aspergillus versicolor*
Chaetomin	Anti-tumorigenic, inhibits mitochondrial respiration and hypoxia-inducible transcription [64,65]	*Chaetomium cochliodes, Chaetomium globosum*
Chaetoglobosin	Cytotoxic cytochalasin, inhibits actin polymerization and glucose transport [45]	*Chaetomium globosum, Penicillium expansum*
Chaetoviridins	Antifungal activity against plant pathogens [66]	*Chaetomium cochliodes, Chaetomium globosum*
Communesins	Neurotoxic [45]	*Penicillium sp.*
Emodin	Cytotoxic, anti-inflammatory, mitochondrial toxin [67]	*Aspergillus sp.*
Melinacidin	Cytotoxic, immunosuppressive [68]	*Agrostalagmus luteoalbus*
Sterigmatocystin	Cytostatic, carcinogenic, inhibits DNA synthesis [67,69]	*Aspergillus versicolor*
Trilongins	Lethal toxins making K^+ and Na^+ ion channels [60]	*Trichoderma sp.*

Supplementary references:

63. Kawai, K.; Nozawa, Y.; Maebayashi, Y.; Yamazaki, M.; Hamasaki, T. Averufin, an anthraquinone mycotoxin possessing a potent uncoupling effect on mitochondrial respiration. *Appl. Environ. Microbiol.* **1984**, *47*, 481–483.
64. Dewangan, J.; Srivastava, S.; Mishra, S.; Pandey, P.K.; Divakar, A.; Rath, S.K. Chetomin induces apoptosis in human triple-negative breast cancer cells by promoting calcium overload and mitochondrial dysfunction. *Biochem. Biophys. Res. Commun.* **2018**, *495*, 1915–1921.
65. Min, S.; Wang, X.; Du, Q.; Gong, H.; Yang, Y.; Wang, T.; Wu, N.; Liu, X.; Li, W.; Zhao, C.; et al. Chetomin, a Hsp90/HIF1α pathway inhibitor, effectively targets lung cancer stem cells and non-stem cells. *Cancer Biol. Ther.* **2020**, *21*, 698–708.
66. Park, J.H.; Choi, G.J.; Jang, K.S.; Lim, H.K.; Kim, H.T.; Cho, K.Y.; Kim, J.C. Antifungal activity against plant pathogenic fungi of chaetoviridins isolated from *Chaetomium globosum*. *FEMS Microbiol. Lett.* **2005**, *252*, 309–313.
67. Mikkola, R.; Andersson, M.A.; Hautaniemi, M.; Salkinoja-Salonen, M.S. Toxic indole alkaloids avrainvillamide and stephacidin B produced by a biocide tolerant indoor mold *Aspergillus westerdijkiae*. *Toxicon* **2015**, *99*, 58–67.
68. Zingales, V.; Fernández-Franzón, M.; Ruiz, M.J. Sterigmatocystin: Occurrence, toxicity and molecular mechanisms of action—A review. *Food Chem. Toxicol.* **2020**, *146*, 111802.
69. Andersson, M.A.; Salo, J.; Mikkola, R.; Marik, T.; Kredics, L.; Kurnitski, J.; Salonen, H. Melinacidin-producing *Acrostalagmus luteoalbus*, a major constituent of mixed mycobiota contaminating insulation material in an outdoor wall. *Pathogens* **2021**, *10*, 843.

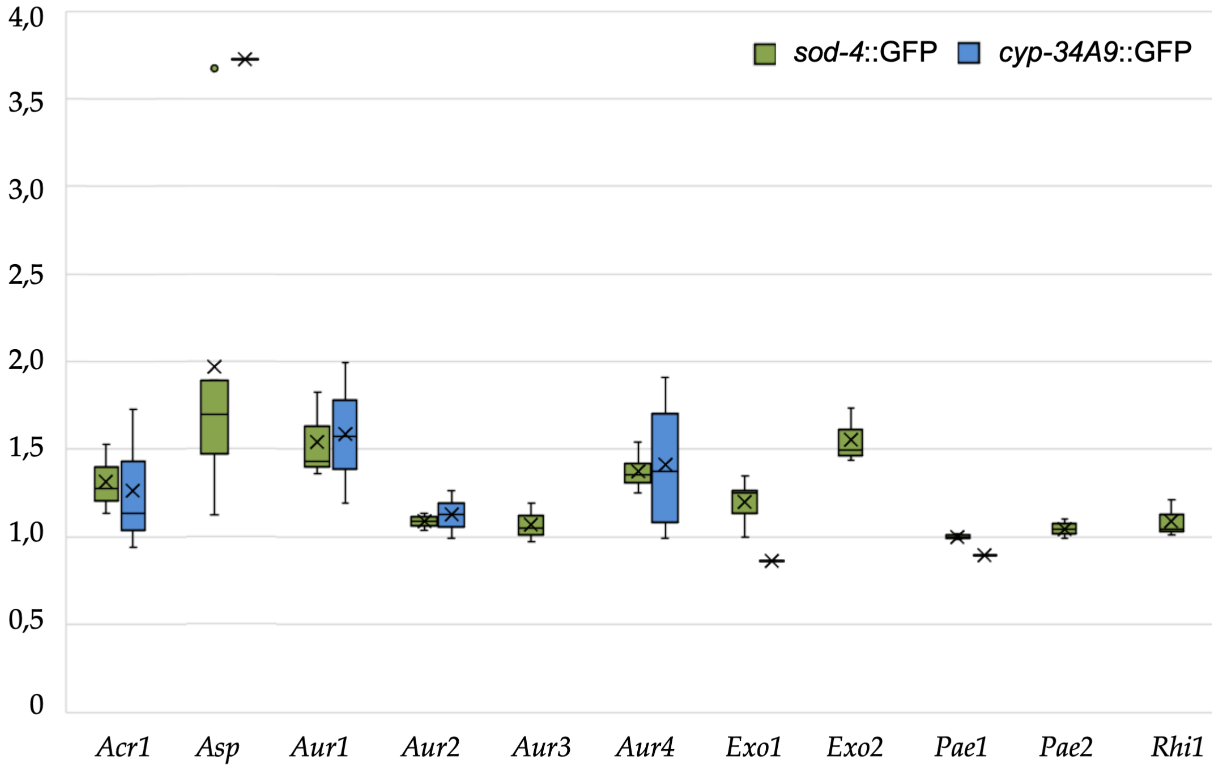

Supplementary Figure 1. Original spectrometric data for Table 3. Shown are box plot data on the effects of biomass dispersals of indoor fungi in *in vivo* bioassays with the *C. elegans sod4*::GFP and *cyp34A9*::GFP strains. Data have been collected from one to four parallel samples.

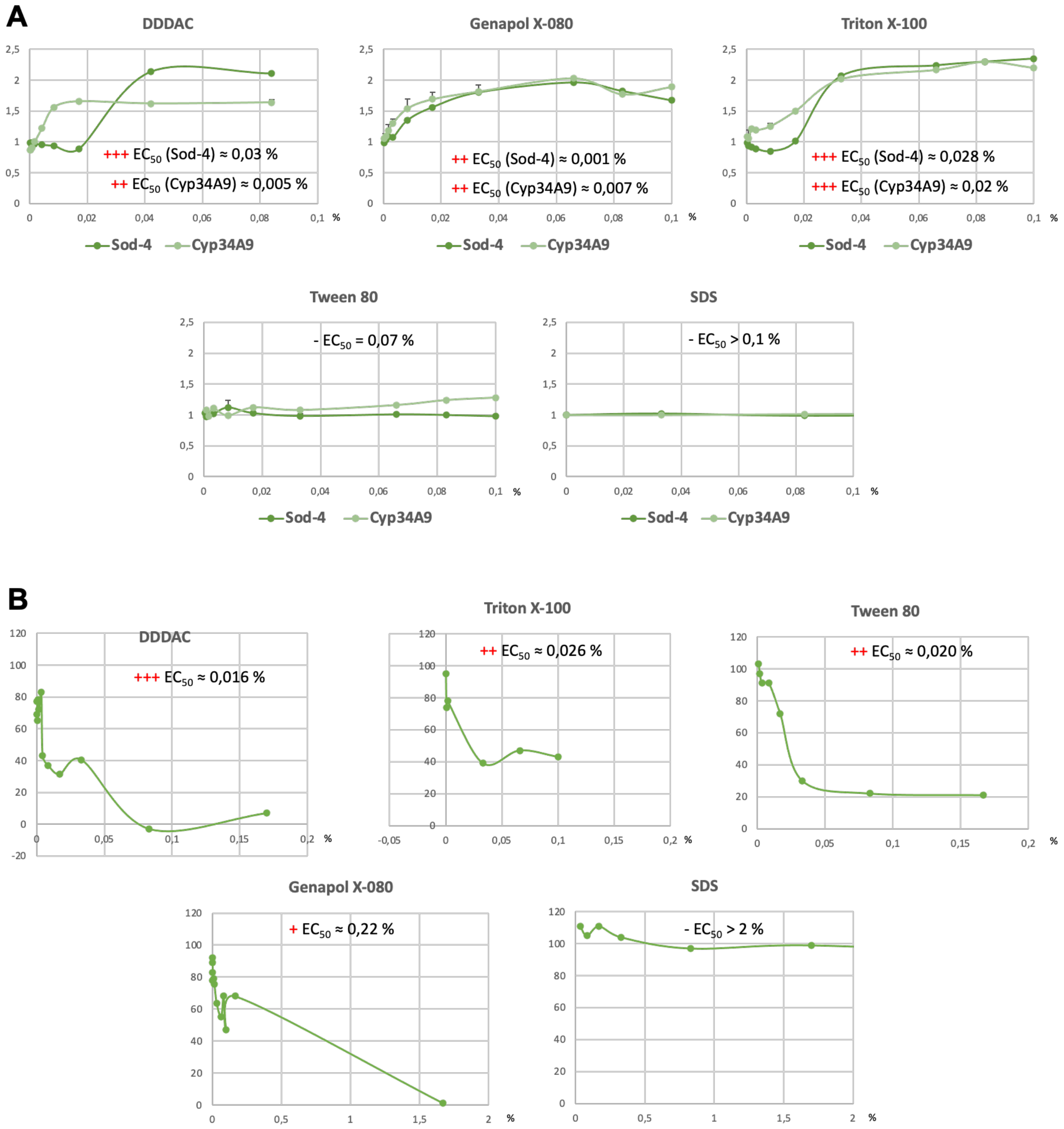

Supplementary Figure 2. Original spectrometric and motility data for Table 4. Dose-dependent effects of chemical surfactants on fluorescent responses (**A**) and motility (**B**) of *C. elegans* reporter strains. EC_{50} refers to a concentration where half maximal effects were observed.
